# Preoperative Oral Carbohydrate Levels in Patients with Type 2 Diabetes Mellitus: The Clinical Guiding Significance of Free Fatty Acids

**DOI:** 10.3389/fsurg.2022.814540

**Published:** 2022-05-26

**Authors:** Jiuhui Yang, Xiangming Ding, Ning Wang, Yujin Pan, Erwei Xiao, Senmao Mu, Liancai Wang, Dongxiao Li, Deyu Li

**Affiliations:** ^1^ Department of Hepatobiliary Pancreatic Surgery, People's Hospital of Zhengzhou University, Henan Provincial People's Hospital, Zhengzhou, China; ^2^ Department of Hepatobiliary Pancreatic Surgery, Henan Provincial People's Hospital, People's Hospital of Zhengzhou University, Zhengzhou, China; ^3^ Department of Gastroenterology, Henan Provincial People's Hospital, People's Hospital of Zhengzhou University, Zhengzhou, China; ^4^ Zhengzhou Key Laboratory of Minimally Invasive Treatment for Liver Cancer, Henan Provincial People's Hospital, Zhengzhou, China; ^5^ Henan Provincial Key Laboratory of Hepatobiliary and Pancreatic Diseases, Henan Provincial People's Hospital, Zhengzhou, China

**Keywords:** preoperative oral carbohydrate drinks, type 2 diabetes mellitus, free fatty acids, insulin resistance, abdominal surgery

## Abstract

**Background:**

It is still controversial whether preoperative oral carbohydrate (POC) should be applied to patients with type 2 diabetes mellitus (T2DM) in the enhanced recovery after surgery (ERAS) protocol. There is no relevant consensus or indicators to provide guidance as to whether T2DM patients should take POC.

**Methods:**

In total, 164 T2DM patients who underwent laparoscopic hepatectomy were analyzed. According to the level of blood free fatty acids (FFAs) and whether the patients received POC, the patients were divided into 6 groups: the low FFA carbohydrate group (LFFAC group), low FFA fasting water group (LFFAF group), medium FFA carbohydrate group (MFFAC group), medium FFA fasting water group (MFFAF group), high FFA carbohydrate group (HFFAC group) and high FFA fasting water group (HFFAF group).

**Results:**

Patients with low FFA levels showed better perioperative blood glucose control and a lower incidence of postoperative complications than those in the medium and high FFA groups, especially when patients received POC. Further analyses revealed that the postoperative plasma concentrations of IL-6 and TNF-α were significantly decreased in the POC group compared with the fasting water group, except for patients with high FFA levels. Receiver operating characteristic (ROC) curve analysis revealed that when the FFA concentration was higher than 0.745 mmol/L, the risk of poor blood glucose control during the perioperative period was increased.

**Conclusions:**

FFAs have clinical guiding significance for the application of POC in patients with T2DM under ERAS administration. T2DM patients with low FFAs are more suitable for receiving POC.

## Introduction

Preoperative oral carbohydrate drinks (POCs) have been widely accepted as one of the enhanced recovery after surgery (ERAS) strategies. However, is still controversial whether POCs should be applied to patients with type 2 diabetes mellitus (T2DM) (1). Some clinicians have claimed that POC neither exerts significant benefits for perioperative glycaemic control nor accelerates ERAS (2–4), and it is recommended to suspend the use of POC for T2DM patients (5, 6). However, supporters of POC believe that intake of carbohydrates 2–3 h before surgery can reduce thirst, hunger, anxiety, insulin resistance, nausea, vomiting and other complications (7–10).

FFAs are the product of adipose tissue lipolysis, which can affect glucose oxidative uptake and gluconeogenesis and ultimately affect insulin secretion and insulin signal transduction (11–13). Some studies have shown that impaired utilization of fatty acids may play a role in the aetiology of insulin resistance in skeletal muscle and liver (11, 14). Foods with high levels of fat, cholesterol and sugar produce metabolites such as lactic acid or glucose instead of lipid energy substrates in the skeletal muscle, which can prevent lipid oxidation and increase intramuscular fat content, thereby reducing muscle uptake and oxidation of FFAs. A decrease in skeletal muscle utilization of FFAs may increase the flow of FFAs to the liver, and the oxidative damage of liver FFAs caused by frequent intake of high fat and high cholesterol food will promote liver fat deposition and insulin resistance (15, 16). We, therefore, wondered whether FFA might function as a potential indicator to guide the application of POC for T2DM patients.

In this study, we retrospectively investigated T2DM patients who underwent laparoscopic hepatectomy in ×××× People᾽s Hospital affiliated to ×××× University to investigate the effect of POC on T2DM patients and to determine the clinical guiding significance of FFA for the application of POC in patients with T2DM.

## Methods

### Ethical Statement

The study was conducted in accordance with the Declaration of Helsinki (as revised in 2013). This retrospective study was approved by the Ethics Committee of ×××× People᾽s Hospital affiliated with ×××× University. A total of 276 T2DM patients who underwent laparoscopic hepatectomy between January 2015 and December 2020 were enrolled in this study. All data were collected from the medical records department of the hospital and analyzed for demographic profile, comorbidities, nutritional status, diagnosis, surgical procedures and morbidity. All patients gave informed consent to surgery as well as the use of their anonymous clinical data for research purposes.

### Inclusion and Exclusion Criteria

Inclusion criteria: (1) Patients diagnosed with T2DM; (2) Preoperative imaging examination (computed tomography/magnetic resonance imaging/positron emission tomography/ultrasound) with two or more examinations supporting the diagnosis of hepatocellular carcinoma or a preoperative pathological diagnosis of hepatocellular carcinoma; (3) Age 18–70 years old; (4) Liver function rating before and after surgery as Class A (Child–Pugh–Turcotte score); (5) Signed written informed consent and agreed to receive surgical treatment.

Exclusion criteria: (1) Preoperative examination showed existing metastasis and only palliative resection was performed. (2) Previous history of malignant tumour; (3) Patients with abnormal gastric emptying or intestinal obstruction; (4) Use of steroids or immunosuppressants; (5) Women during pregnancy or lactation.

### Grouping Method

After applying the inclusion and exclusion criteria, 164 patients were selected from among 276 T2DM patients. Twenty-eight patients in the LFFA group receiving POC were placed in the low FFA carbohydrate group (LFFAC group), and 20 patients who were forbidden to consume anything orally before the operation were placed in the low FFA fasting water group (LFFAF group). The patients in the MFFA and HFFA groups were classified as described in the next section. There were 35 patients in the medium free fatty acid carbohydrate group (MFFAC group), 37 patients in the medium free fatty acid fasting group (MFFAF group), 23 patients in the high free fatty acid carbohydrate group (HFFAC group) and 21 patients in the high free fatty acid fasting group (HFFAF group).

### General Information

A retrospective analysis of 164 T2DM patients who underwent laparoscopic hepatectomy in the Department of Hepatobiliary Surgery, People᾽s Hospital of ×××× University, from January 2015 to December 2020, was conducted, and their free fatty acid levels were evaluated. According to the first quartile and the third quartile, the patients were divided into three groups: the low free fatty acid (LFFA) group, medium free fatty acid (MFFA) group and high free fatty acid (HFFA) group.

### Administration of POC

The standard POC method in our centre is to administer an oral carbohydrate solution 400 mL (containing 7.5 g carbohydrate per 100 mL) the night before surgery from 21:00 p.m. to 24:00 p.m. and another 300 mL 2 h prior to surgery.

### Data Processing

SPSS 26.0 software was used for data processing. All measurement data were tested by Shapiro–Wilk normality, and the data conforming to a normal distribution were expressed as the mean ± standard deviation. Variance analysis was used among multiple groups, and an independent sample t-test was used between two groups. The data that did not conform to a normal distribution were subjected to the Kruskal–Wallis test. The enumeration data were expressed as percentages, and the *χ*^2^ test was used.

## Results

### General Information Comparison

As shown in Table 1, there were no significant differences in sex, age, body mass index (BMI), preoperative glycosylated haemoglobin, operation time, American Society of Anaesthesiologists (ASA) score or complications among the six groups (*p* > 0.05).

**Table 1:** General information of each group.

Clinicopathological variables	LFFAC (*n* = 28)	LFFAF (*n* = 20)	MFFAC (*n* = 35)	MFFAF (*n* = 37)	HFFAC (*n* = 23)	HFFAF (*n* = 21)	*p*
Gender [Percent (*n*)]							0.939
Male	53.6% (15)	55.0% (11)	60.0% (21)	48.6% (18)	52.2% (12)	47.6% (10)	
Female	46.4% (13)	45.0% (9)	40.0% (14)	51.4% (19)	47.8% (11)	52.4% (11)	
Age (year)	59.89 [12.41]	55.20 [12.10]	60.46 [11.16]	59.35 [11.83]	62.57 [11.67]	59.05 [11.97]	0.548
BMI (kg/m^2^)	25.42 [1.16]	25.17 [1.12]	25.31 [0.86]	25.35 [1.27]	25.22 [0.76]	25.28 [0.72]	0.939
HbA1c (%)	7.18 [0.74]	7.09 [0.94]	7.40 [1.02]	7.12 [0.86]	7.25 [0.80]	6.83 [0.63]	0.993
Operation time (minutes)	165.92 [34.13]	155.90 [20.06]	161.17 [13.27]	163.14 [30.20]	153.78 [20.57]	158.86 [27.81]	1.000
ASA score [Percent (*n*)]							0.957
1	39.3% (11)	25.0% (5)	37.1% (13)	37.8% (14)	43.5% (10)	42.9% (9)	
2	39.3 (11)	40.0% (8)	37.1% (13)	37.8% (14)	39.1% (9)	42.9% (9)	
3	21.4% (6)	35.0% (7)	25.8% (9)	24.4% (9)	17.4% (4)	14.2% (3)	
Comorbidities [Percent (*n*)]							0.913
Viral hepatitis B	39.3% (11)	45.0% (9)	34.3% (12)	40.5% (15)	39.1% (9)	38.1% (8)	
COPD	14.3% (4)	15.0% (3)	14.3% (5)	13.5% (5)	17.4% (4)	9.5% (2)	
Hypertension	17.9% (5)	15.0% (3)	8.6% (3)	10.8% (4)	8.7% (2)	9.5% (2)	
Heart disease	14.3% (4)	10.0% (2)	11.4% (4)	10.8% (4)	4.3% (1)	19.0% (4)	

*BMI, body mass index; HbA1c, haemoglobin A1c; ASA, American Society of Anaesthesiologists; COPD, chronic obstructive pulmonary disease. The standard deviations of BMI, HbA1c and operation time are shown in square brackets.*

### Perioperative Blood Glucose Analysis

**Figure 1:**
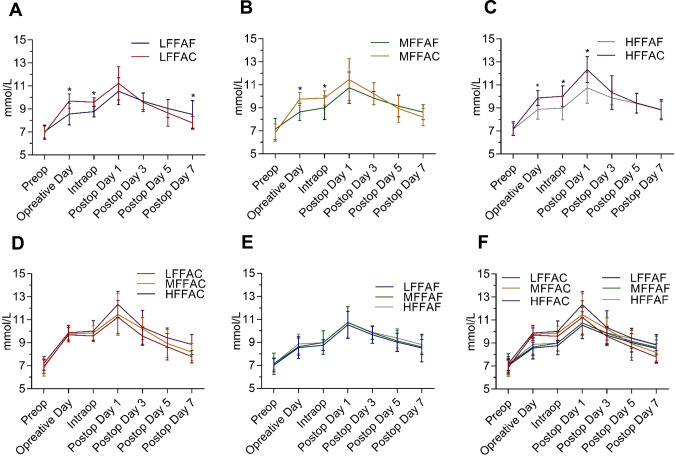
Perioperative glycaemic control timeline in each indicated group during the perioperative period. (**A**) Comparison of perioperative blood glucose between the low FFA carbohydrate group (LFFAC group) and the low FFA fasting water group (LFFAF group). (**B**) Comparison of perioperative blood glucose between the medium FFA carbohydrate group (MFFAC group) and the medium FFA fasting water group (MFFAF group). (**C**) Comparison of perioperative blood glucose between the high FFA carbohydrate group and the high FFA fasting water group. (**D**,**E**)Comparison of perioperative blood glucose in patients with different FFA values under POC or fasting water conditions. (**F**)Comparison of perioperative blood glucose among the 6 groups. *, represents a significant difference (*p* < 0.05).

Previous studies have shown that FFA is associated with insulin resistance (7–9); therefore, we asked whether FFA might function as a potential indicator to guide T2DM patients to use POC. The preoperative, operative, intraoperative, postoperative Day 1, postoperative Day 3, postoperative Day 5 and postoperative Day 7 blood glucose values were analyzed and are shown in Figure 1. None of the patients altered their diabetes management during the perioperative period.

To investigate whether POC could alleviate postoperative insulin resistance, we compared the level of perioperative blood glucose between the POC and the fasting water groups; however, there was no significant difference in postoperative blood glucose between these two groups (Supplementary Figure S1). We further analyzed the perioperative blood glucose concentrations between the POC and the fasting water groups in patients with low, medium and high FFA levels. In comparison with the LFFAF group, the blood glucose level in the LFFAC group was higher on the day of the operation; however, it dropped quickly after the surgery and was significantly lower than that in the LFFAF group on the 7th day after the operation (Figure 1A). Although the preoperative blood glucose level of the MFFAC group was significantly higher than that in the corresponding fasting water group, the postoperative blood glucose was lower than that in the MFFAF group, but there was no significant difference (Figure 1B). Notably, in patients with high FFAs, POC dramatically increased the blood glucose value on the operative day and on intraoperative and postoperative Day 1, and the level of postoperative blood glucose continued to be higher than that in the fasting water group (Figure 1C).

Next, we compared the level of perioperative blood glucose among patients with low, medium and high FFAs under POC or fasting water conditions. We found that patients with low or medium FFAs showed relatively better preoperative blood glucose control in both the POC and the fasting water condition (Figures 1D,E). Within the 6 groups, the LFFAC group exhibited the best perioperative blood glucose control, while the HFFAC group exhibited the worst perioperative blood glucose control (Figure 1F). Collectively, these observations indicated that T2DM patients with low FFAs had better preoperative blood glucose control, especially when patients received POC.

### Postoperative Complications

No statistically significant differences were found in the surgical procedures among the six groups (Supplementary Table S2). The postoperative complications in each group are recorded in Table 2. We compared the incidence of postoperative complications between the POC and the fasting water groups; however, there was no significant difference between these two groups (Supplementary Table S1). We further analyzed the incidence of postoperative complications between POC and fasting water conditions in patients with low, medium and high FFA levels. Statistical analyses showed that the incidence of postoperative wound infection in the LFFAC group was lower than that in the corresponding fasting water group (*p* < 0.05), while the incidence of postoperative wound infection in the HFFAC group was higher than that in the HFFAF group (*p* < 0.05) (Table 3).

**Table 2:** Postoperative complications of each group.

Postoperative complications	LFFAC (*n* = 28)	LFFAF (*n* = 20)	MFFAC (*n* = 235)	MFFAF (*n* = 37)	HFFAC (*n* = 23)	HFFAF (*n* = 21)
Wound infection	0	4	6	8	14	6
Pulmonary infection	1	2	5	5	6	4
Pleural effusion	1	4	4	5	5	2
Peritoneal effusion	0	1	3	4	5	3
Venous thrombosis	1	1	3	4	2	2
Biliary fistula	0	0	1	2	2	1
Intra-abdominal haemorrhage	0	0	0	0	1	1
Count of postoperative complications^a^						
Zero	26	9	19	18	3	6
One	2	10	11	11	9	11
Two	0	1	4	7	9	4
Three	0	0	1	1	1	0
Four	0	0	0	0	1	0

^a^ *Includes postoperative complications: wound infection, pulmonary infection, pleural effusion, venous thrombosis, biliary fistula, intra-abdominal haemorrhage.*

**Table 3:** *P* value among the indicated groups.

	Incision infection	Pulmonary infection	Pleural effusion	Peritoneal effusion	Venous thrombosis	Bile fistula	Intra-abdominal haemorrhage
LFFAC/LFFAF	0.025^a^	0.373	0.088	0.417	0.665	N/A	N/A
MFFAC/MFFAF	0.429	0.596	0.536	0.532	0.532	0.521	N/A
HFFAC/HFFAF	0.032^a^	0.424	0.264	0.404	0.661	0.535	0.535
LFFAC/HFFAC	0.000^a^	0.027^a^	0.058	0.014^a^	0.425	0.198	0.198

*^a^* *Represents a significant difference (p < 0.05).*

To further investigate the effect of FFAs on postoperative complications in patients receiving POC, the postoperative complications between LFFAC and HFFAC patients were compared. We found that the incidence of postoperative wound infection, pulmonary infection and peritoneal effusion in the LFFAC group was significantly lower than that in the HFFAC group (Table 3). Collectively, these observations suggested that T2DM patients with low FFAs had a lower incidence of postoperative complications, especially when patients received POC.

### Insulin Resistance–Related Factors

**Figure 2:**
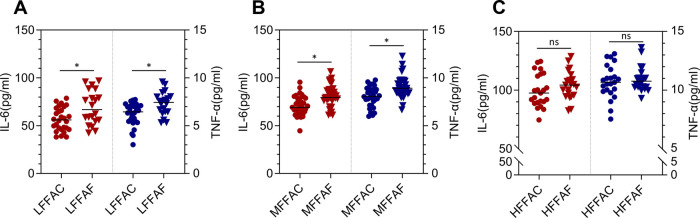
Plasma IL-6 and TNF-α levels on postoperative Day 1. (**A**) Comparison of IL-6 and TNF-α levels between LFFAC and LFFAF. (**B**) Comparison of IL-6 and TNF-α between MFFAC and MFFAF. (**C**) Comparison of IL-6 and TNF-α levels between HFFAC and HFFAF. * represents a significant difference (*p* < 0.05).

Previous studies have revealed that interleukin-6 (IL-6) and tumour necrosis factor-α (TNF-α) are involved in insulin resistance (17, 18); therefore, we explored the clinical significance between these insulin resistance-related factors and FFAs. We first compared the concentrations of IL-6 and TNF-α on the first day after surgery between POC and fasting water conditions in patients with low, medium and high FFA levels. In patients with low or medium FFA levels, the concentrations of IL-6 and TNF-α were substantially reduced in the POC group compared with the fasting water group (Figures 2A,B), while there were no obvious changes in the concentrations of IL-6 and TNF-α between the HFFAC group and the HFFAF group (Figure 2C).

**Figure 3:**
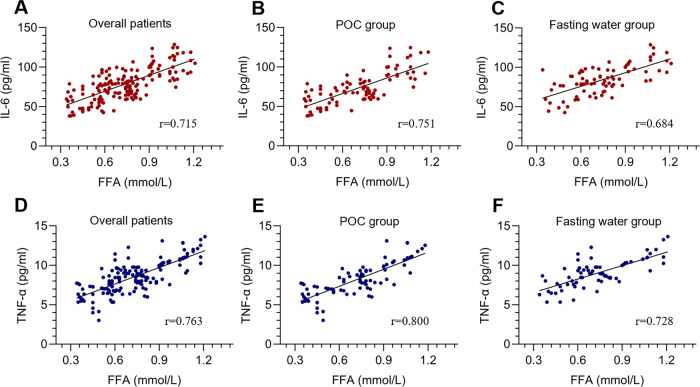
Correlation analysis between FFA and IL-6 or TNF-α. The r refers to the Pearson correlation coefficient.

We further analyzed the Pearson correlation between FFA and postoperative IL-6 or TNF-α. The results revealed that there was a linear correlation between FFA and IL-6 or TNF-α in the overall patients as well as the POC and fasting water group (Figure 3), and the correlation was stronger in patients taking POC than in the overall patients and fasting water group (Figures 3C,F). Collectively, these observations indicated that the better preoperative blood glucose control of patients with low FFAs might partly be due to a reduction of IL-6 and TNF-α.

### Receiver Operating Characteristic Curve of Poor Glycaemic Control

**Figure 4:**
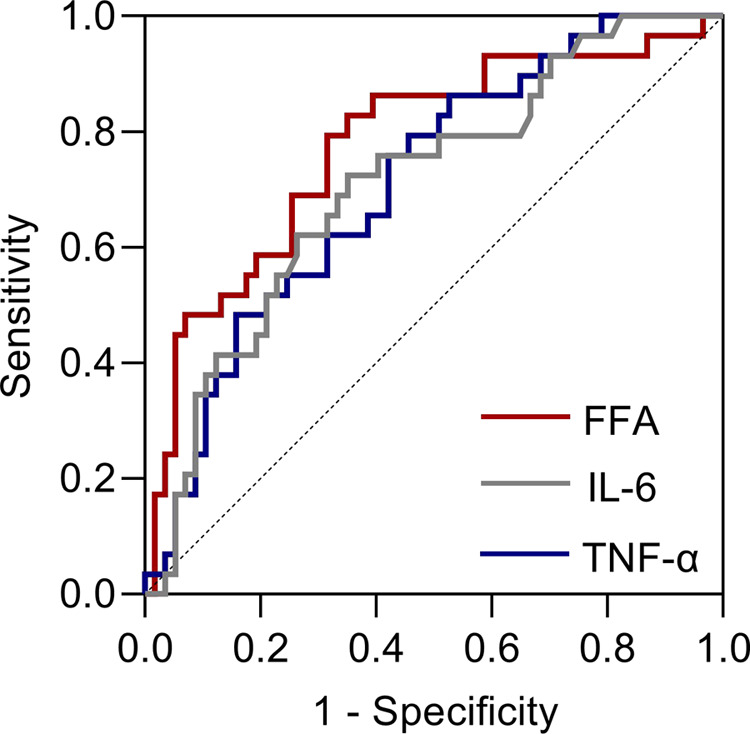
Receiver operating characteristic (ROC) curves for poor glycaemic control after taking POC.

According to the *Chinese clinical practice guidelines for perioperative blood glucose management*, patients with postoperative blood glucose exceeding 12.0 mmol/L are considered to have poor blood glucose control during the perioperative period (19). We, therefore, examined the receiver operating characteristic (ROC) curve for poor glycaemic control to visualize the trade-off between sensitivity and specificity (Figure 4). The area under the curve (AUC) values of FFA, IL-6 and TNF-α for poor blood glucose control during the perioperative period were 0.776, 0.711 and 0.709, respectively. When FFA (mmol/L) > 0.745, IL-6 (pg/mL) > 69.400 and TNF-α (pg/mL) > 7.975, the risk of poor blood glucose control during the perioperative period was higher.

## Discussion

Our research aimed to develop clinical guidance for the application of POC to T2DM patients during ERAS. In this study, we demonstrated that patients with low FFA levels showed better perioperative blood glucose control and a lower incidence of postoperative complications than those in the medium and high FFA groups, especially when patients received POC. The postoperative plasma concentrations of IL-6 and TNF-α were significantly decreased in the POC group compared with the fasting water group, except for patients with high FFA levels. Viganò, Cereda *et al*. showed that POC could reduce postoperative inflammatory markers, which is consistent with our research results (20).

One of the theories of postoperative insulin resistance is that trauma affects the PI3K-PDK-1-PKB pathway, thereby affecting glucose transport, oxidative metabolism and glycogen synthesis (21, 22). Studies have shown that postoperative IL-6 and TNF-α values are positively correlated with insulin resistance (17, 18). TNF-α plays an important role in this process since it can block insulin signal transduction by inhibiting tyrosine protein kinase (TPK) activity (23–25). Animal experiments showed that after the application of soluble TNF-α receptor IgG to neutralize TNF-α, insulin-stimulated receptor autophosphorylation and insulin receptor substrate-1 (IRS-1) phosphorylation in the rat skeletal muscle and adipose tissue were greatly improved, and tissue sensitivity to insulin was increased (26, 27). Increased IL-6 inhibits insulin signalling and leads to insulin resistance, mainly in the liver (27, 28). Chronic exposure to IL-6 selectively induces insulin resistance in the liver. Acute IL-6 elevation can induce the expression of suppressor of cytokine signalling-3 (SOCS-3). Overexpression of SOCS-3 has been shown to impair insulin-dependent insulin receptor autophosphorylation, IRS-1 tyrosine phosphorylation, phosphatidylinositol 3-kinase association with IRS-1 and protein kinase B (PKB) activation in hepatocytes (29).

The mechanisms by which FFA leads to insulin resistance remain unclear. Previous studies revealed that the production of lipid metabolites (diacylglycerol), proinflammatory cytokines (TNF-a, IL-1b, IL-6, monocyte chemoattractant protein-1) and cellular stress, including oxidative and endoplasmic reticulum stress, may contribute to FFA-induced insulin resistance (13, 14). Our correlation analysis revealed that that there was a linear correlation between FFA and IL-6 or TNF-α, and the correlation was stronger in patients taking POC. These observations indicated that the better preoperative blood glucose control of patients with low FFAs might in part be due to the reduction of IL-6 and TNF-α.

The ROC curve of poor glycaemic control showed that FFAs had higher sensitivity and specificity than IL-6 and TNF-α in predicting poor perioperative glycaemic control in T2DM patients. In addition, considering that the FFA values were measured before the operation while IL-6 and TNF-α were measured on the first day after the operation, we suggest that FFAs have higher clinical guiding significance than IL-6 or TNF-α for the application of POC in patients with T2DM.

In conclusion, FFAs have clinical guiding significance for the application of POC in patients with T2DM under ERAS administration. T2DM patients with low FFAs are more suitable for receiving POC.

## Data Availability

The raw data supporting the conclusions of this article will be made available by the authors, without undue reservation.
