# Supplementary figures and images for: Preoperative Oral Carbohydrate Levels in Patients with Type 2 Diabetes Mellitus: The Clinical Guiding Significance of Free Fatty Acids
Source: Front Surg. 2022 May 26;9:814540. doi: 10.3389/fsurg.2022.814540 (PMC9195184; doi:10.3389/fsurg.2022.814540)

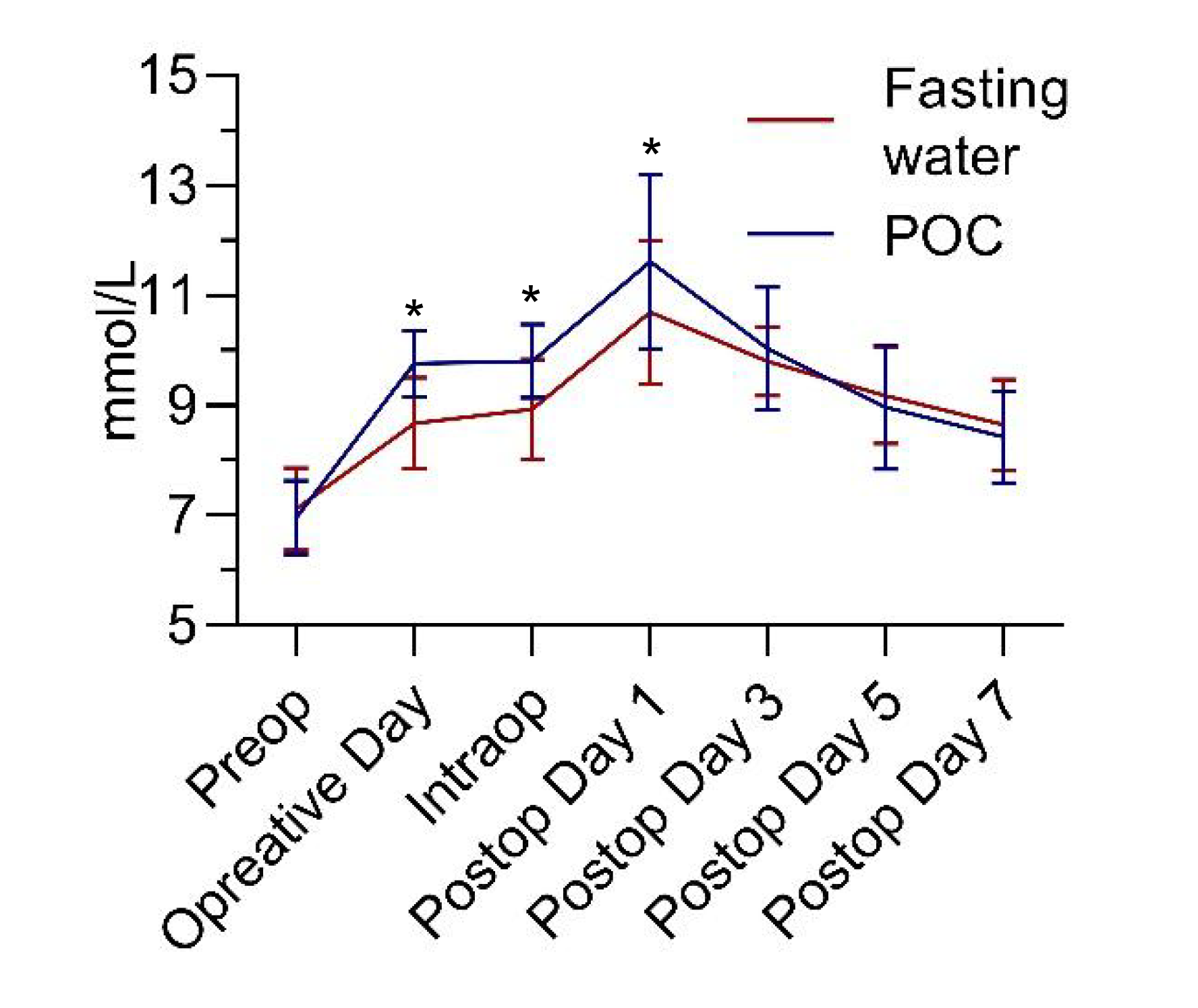

Supplement: Supplementary file 1 [file Figure_5_v1.tif]
