# Supplementary material for: Preoperative Oral Carbohydrate Levels in Patients with Type 2 Diabetes Mellitus: The Clinical Guiding Significance of Free Fatty Acids
Source: Front Surg. 2022 May 26;9:814540. doi: 10.3389/fsurg.2022.814540 (PMC9195184; doi:10.3389/fsurg.2022.814540)
Supplement: Supplementary file 2 [file Table_4_v1.docx]

TABLE S1. Postoperative complications between POC and fasting water group

|  | POC group (86) | Fasting group (78) | P value |
| --- | --- | --- | --- |
| Incision infection | 16 | 18 | 0.480 |
| Pulmonary infection | 12 | 11 | 0.978 |
| Pleural effusion | 10 | 11 | 0.636 |
| Peritoneal effusion | 8 | 8 | 0.837 |
| Venous thrombosis | 6 | 7 | 0.636 |
| Bile fistula | 3 | 3 | 0.903 |
| Intra-abdominal hemorrhage | 2 | 1 | 0.613 |
| Count of postoperative complication* |  |  | 0.204 |
| Zero | 49 | 33 |  |
| One | 22 | 32 |  |
| Two | 12 | 12 |  |
| Three | 2 | 1 |  |
| Four | 1 | 0 |  |

*, includes postoperative complication, incision infection, pulmonary infection, pleural effusion, venous thrombosis, bile fistula, intra-abdominal hemorrhage.
