# Supplementary material for: Preoperative Oral Carbohydrate Levels in Patients with Type 2 Diabetes Mellitus: The Clinical Guiding Significance of Free Fatty Acids
Source: Front Surg. 2022 May 26;9:814540. doi: 10.3389/fsurg.2022.814540 (PMC9195184; doi:10.3389/fsurg.2022.814540)
Supplement: Supplementary file 3 [file Table_5_v1.docx]

TABLE S2. Type of hepatectomy in each group

| Type of hepatectomy | LFFAC (n=28) | LFFAF (n=20) | MFFAC (n=235) | MFFAF (n=37) | HFFAC (n=23) | HFFAF (n=21) | *P* |
| --- | --- | --- | --- | --- | --- | --- | --- |
| Enucleation of hepatic tumour | 3 | 3 | 5 | 6 | 3 | 5 | 0.921 |
| Local hepatectomy | 3 | 5 | 5 | 8 | 2 | 4 |  |
| Hepatic segmentectomy | 14 | 9 | 16 | 10 | 12 | 7 |  |
| Left hemi-hepatectomy | 5 | 1 | 6 | 7 | 4 | 2 |  |
| Right hemi-hepatectomy | 3 | 2 | 3 | 6 | 2 | 3 |  |
